# Supplementary material for: Identification of Biomarkers Related to M2 Macrophage Infiltration in Alzheimer’s Disease
Source: Cells. 2022 Aug 1;11(15):2365. doi: 10.3390/cells11152365 (PMC9367736; doi:10.3390/cells11152365)
Supplement: Supplementary file 1 [file cells-11-02365-s001.zip › Supplementary Figure S1-S3.pdf]

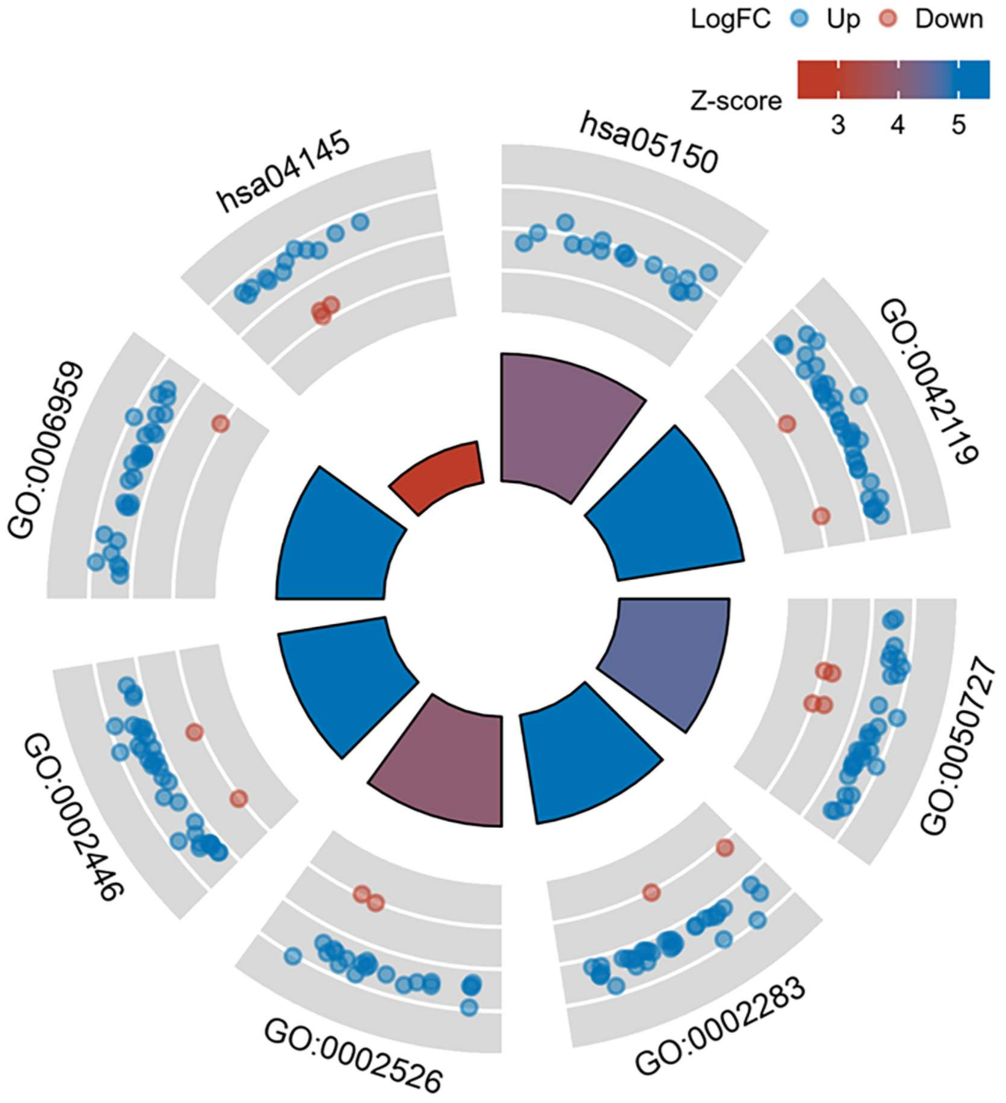

Figure S1. Circos plot, visualization of GO and KEGG results. The inner ring is a bar plot where the height of the bar indicates the significance of the term (−log10 adjusted P-value), and color corresponds to the z-score. The outer ring displays scatterplots of the expression levels (logFC) for the genes in each term.

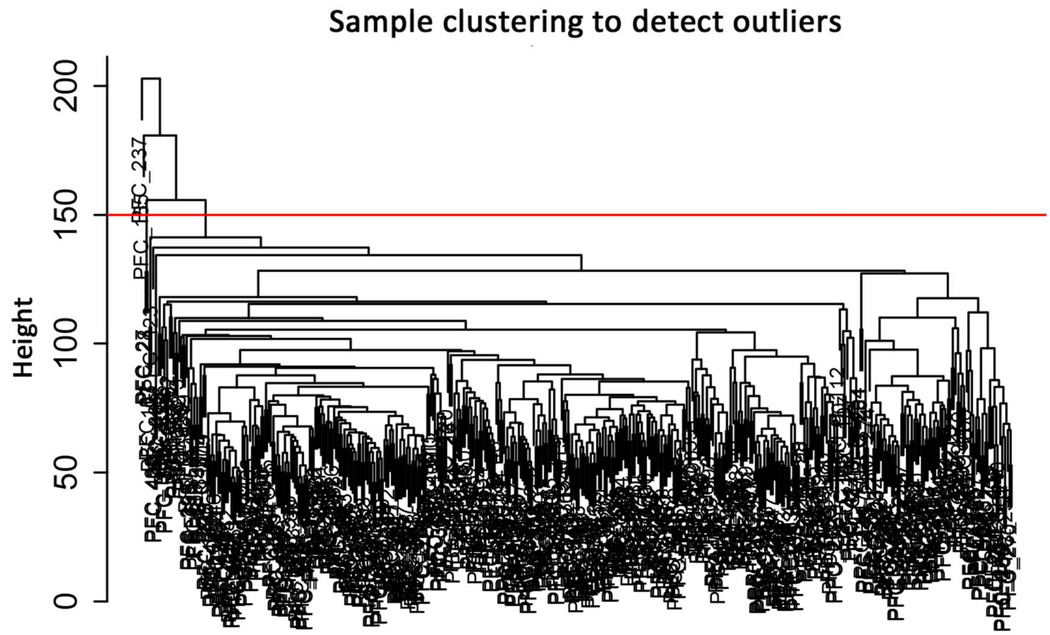

Figure S2. Sample clustering to detect outliers. Cut height 150 and mini sample size 10.

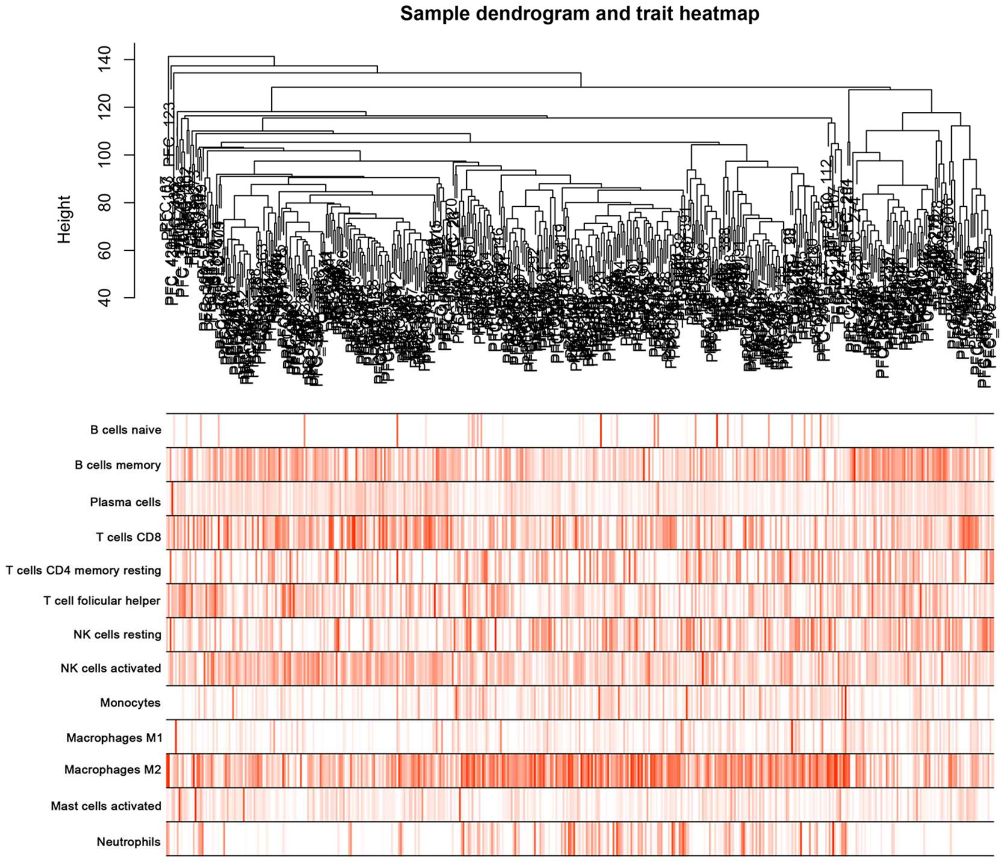

Figure S3. The sample dendrogram and trait heatmap.
